# P01.03. A machine classification measure of meditation ability

**DOI:** 10.1186/1472-6882-12-S1-P3

**Published:** 2012-06-12

**Authors:** A McCollough, K Hild, H Wabeh, B Oken

**Affiliations:** 1 Oregon Health & Science University, Portland, USA

## Purpose

The purpose of the study was to develop an objective measure of meditation quality based on the machine classification of multiple simultaneous physiological recordings.

## Methods

We recorded 32-channel EEG, electro-oculogram (EOG), ECG, respiration, and movement from 12 experienced (mean=13.3 years of practice) Zen Buddhist meditators and 12 non-meditator controls under two conditions for each group: listening to an audiotape and either loving-kindness meditation (meditators) or sitting quietly (non-meditators). The data were split into 15 minute audiotape and meditation epochs. Data were further divided into training and classification sets, and the support vector machine (SVM)-light algorithm was trained on data from each subject.

## Results

Performance of the SVM classifier is measured as the mean AUC for the receiver operating characteristic on the classification set. Perfect separation is AUC =1.0, whereas chance classification is AUC = 0.5. The best performing feature set across subjects was the respiration signal, AUC = 0.90. The EEG (based on the 7 common artifact-free channels) and EOG classification performance had mean AUC values of 0.85 and 0.77, respectively. Frequency domain features analyzed included alpha band (mean AUC 0.54) and scalp EMG (mean AUC of 0.68).

## Conclusion

The classifier was able to reliably separate meditating and non-meditating states using the physiological measures. We were also able to construct a preliminary performance hierarchy of response variables: respiration, EEG, EOG, EMG, and alpha power. The probability of classification can be interpreted as a measure of meditation ability by using the trained classifier to predict class membership in novice meditators. In summary, we have demonstrated a proof-of-concept objective measure of meditation quality.

